# Supplementary material for: Predicting visual function by interpreting a neuronal wiring diagram
Source: Nature. 2024 Oct 2;634(8032):113–23. doi: 10.1038/s41586-024-07953-5 (PMC11446822; doi:10.1038/s41586-024-07953-5)
Supplement: Supplementary file 6 — CRF and ERF predictions for individual TmY4 and TmY9 cells. Analogous to Supplementary Data 3, but for TmY target types. Shown are the top four monosynaptic pathways, the strongest pathway passing through each of the top ten intermediary types (ranking from Extended Data Fig. 7), and the trisynaptic pathway Tm1–TmY–Dm3–TmY (see the section entitled Prediction of spatial normalization). [file 41586_2024_7953_MOESM6_ESM.zip › DataS4/TmY9qΓèÑ/720575940635353195.pdf]

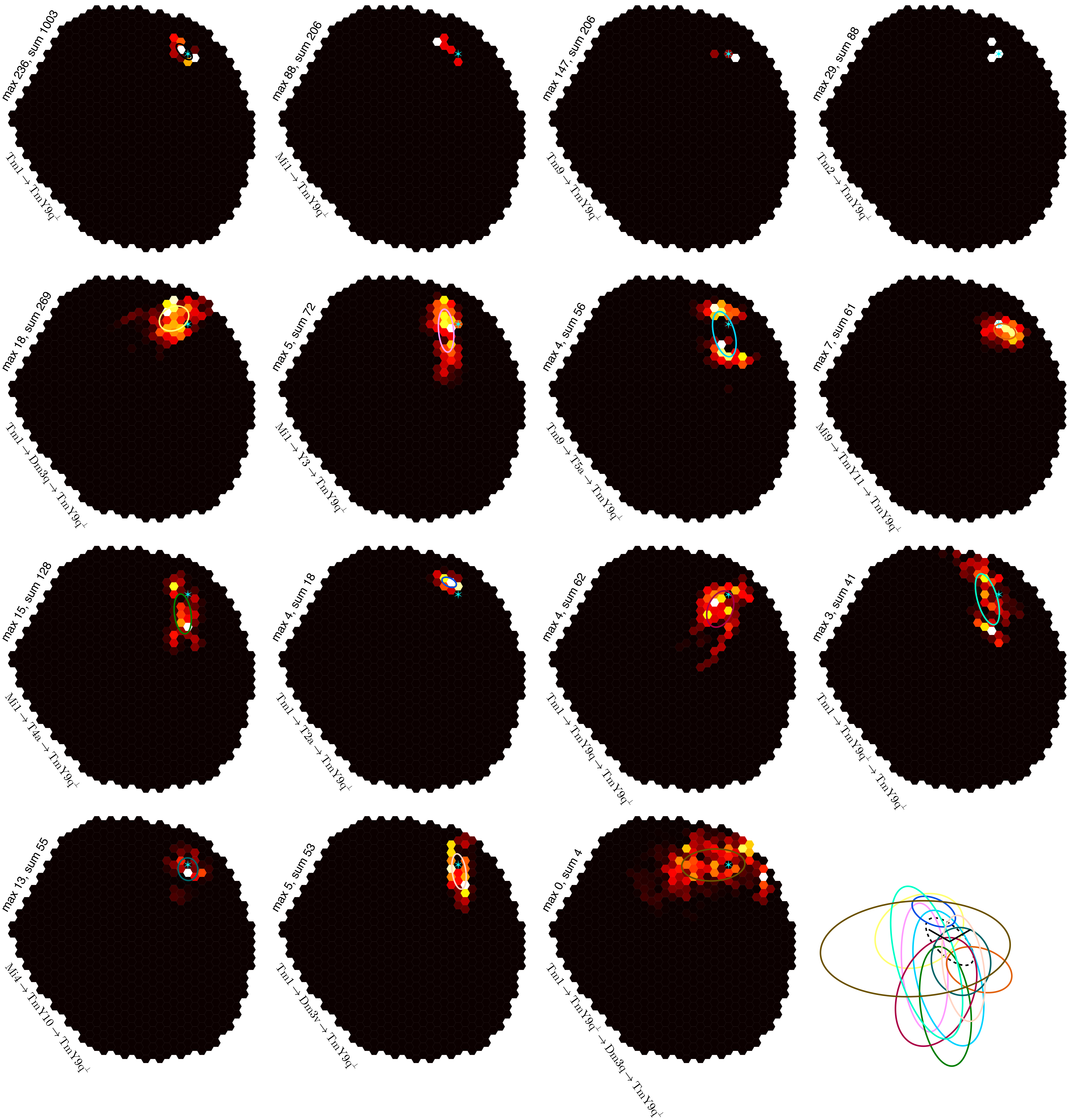
max 236, sum 1003
Tm1 → TmY9q⊥
max 88, sum 206
Mi1 → TmY9q⊥
max 147, sum 206
Tm9 → TmY9q⊥
max 29, sum 88
Tm2 → TmY9q⊥
max 18, sum 269
Tm1 → Dm3q → TmY9q⊥
max 5, sum 72
Mi1 → Y3 → TmY9q⊥
max 4, sum 56
Tm9 → T5a → TmY9q⊥
max 7, sum 61
Mi9 → TmY11 → TmY9q⊥
max 15, sum 128
Mi1 → T4a → TmY9q⊥
max 4, sum 18
Tm1 → T2a → TmY9q⊥
max 4, sum 62
Tm1 → TmY9q → TmY9q⊥
max 3, sum 41
Tm1 → TmY9q⊥ → TmY9q⊥
max 13, sum 55
Mi4 → TmY10 → TmY9q⊥
max 5, sum 53
Tm1 → Dm3v → TmY9q⊥
max 0, sum 4
Tm1 → TmY9q⊥ → Dm3q → TmY9q⊥
